# Distribution of *Bartonella henselae* Variants in Patients, Reservoir Hosts and Vectors in Spain

**DOI:** 10.1371/journal.pone.0068248

**Published:** 2013-07-09

**Authors:** Horacio Gil, Raquel Escudero, Inmaculada Pons, Manuela Rodríguez-Vargas, Coral García-Esteban, Isabel Rodríguez-Moreno, Cristina García-Amil, Bruno Lobo, Félix Valcárcel, Azucena Pérez, Santos Jiménez, Isabel Jado, Ramón Juste, Ferrán Segura, Pedro Anda

**Affiliations:** 1 Centro Nacional de Microbiología, Instituto de Salud Carlos III, Majadahonda, Madrid, Spain; 2 Servei de Malalties Infeccioses, Corporació Sanitària Parc Taulí, Sabadell, Barcelona, Spain; 3 Centro de Investigación en Sanidad Animal, Instituto Nacional de Investigación y Tecnología Agraria y Alimentaria, Valdeolmos, Madrid, Spain; 4 Consejería de Salud, Consumo y Bienestar Social de La Rioja, Gobierno de La Rioja, Logroño, La Rioja, Spain; 5 Sanidad Animal, Neiker-Tecnalia, Instituto Vasco de Investigación y Desarrollo Agrario, Derio, Vizcaya, Spain; University of Exeter Medical School, United Kingdom

## Abstract

We have studied the diversity of *B. henselae* circulating in patients, reservoir hosts and vectors in Spain. In total, we have fully characterized 53 clinical samples from 46 patients, as well as 78 *B. henselae* isolates obtained from 35 cats from La Rioja and Catalonia (northeastern Spain), four positive cat blood samples from which no isolates were obtained, and three positive fleas by Multiple Locus Sequence Typing and Multiple Locus Variable Number Tandem Repeats Analysis. This study represents the largest series of human cases characterized with these methods, with 10 different sequence types and 41 MLVA profiles. Two of the sequence types and 35 of the profiles were not described previously. Most of the *B. henselae* variants belonged to ST5. Also, we have identified a common profile (72) which is well distributed in Spain and was found to persist over time. Indeed, this profile seems to be the origin from which most of the variants identified in this study have been generated. In addition, ST5, ST6 and ST9 were found associated with felines, whereas ST1, ST5 and ST8 were the most frequent sequence types found infecting humans. Interestingly, some of the feline associated variants never found on patients were located in a separate clade, which could represent a group of strains less pathogenic for humans.

## Introduction

*Bartonella henselae* is a facultative intracellular alphaproteobacterium that can infect the erythrocytes and the endothelial cells of their hosts [1], [2]. The major reservoir for this zoonotic pathogen is the feline population. The cat flea (*Ctenocephalides felis*) plays a vector role, transmitting this agent among cats [3]. Flea feces are the main source of infection and can be inoculated by contaminated cat claws to other cats or accidentally to humans [4].

*B. henselae* is the etiological agent of cat scratch disease (CSD), whose main symptom is a regional lymphadenopathy that may be accompanied by other manifestations such as fever or fatigue [5], [6]. This disease is self-limiting and mainly produces a regional affectation, although dissemination can occur, leading to other symptoms such as bacteremia, neuroretinitis, endocarditis or neurological manifestations [5], [6]. The immunological status of the patient can also influence the presentation of the disease; immunocompromised patients can present multi-vasoproliferative lesions in the liver or spleen (visceral peliosis), or on the skin (bacillary angiomatosis) [5], [6].

The diversity of *B. henselae* has been assessed by different characterization methods. Based on the 16S rRNA, two main genotypes were identified [7], [8]. Genotype (GT) II was detected most often in cats from USA, Europe and Australia [9]–[12], while GT I was predominant in cats from Asia [13]–[17]. Also, GT I seemed to be more frequently associated with infection in humans [11], [12], [18]. Subsequently, a multi-locus sequence typing (MLST) scheme was described, presenting a larger discriminatory power than 16S rRNA [11], [19], [20]. To date, this method has differentiated 359 isolates and clinical samples into three main clonal complexes and 30 sequence types (ST). MLST reveals that some STs are more associated with human disease such as ST1, ST2, ST5 and ST8, while ST6 and ST7 are mainly found in felines [11], [21], [22]. Additionally, a multispacer typing method was found to be more discriminatory than MLST [23]. This method was used to group 252 specimens in four separate lineages and 57 STs. Those STs which infect humans fall in lineages 1, 2 and 3, while lineage 4 is composed exclusively of feline isolates [24], [25]. Finally, multi-locus variable number tandem repeats (VNTR) analysis (MLVA) is the method with the highest discriminatory power, with 174 different profiles described until now after the analysis of 481 isolates [12], [26], [27]. MLVA organized the profiles in two main clades, placing all the human isolates in one of them [12]. Comparing the different typing methods, 16S rRNA genotyping was not entirely congruent with the cluster distribution observed with other methods [12], [19], [25]. Regarding MLST, this method seemed initially to be in accordance with the structure defined by the multispacer typing [23], although more recent studies have found discrepancies in the clusters that contain most of the human-associated genotypes [25], [28]. Regarding MLVA, there are scarce data for comparison due to the lack of studies or strains characterized by different typing methods. Therefore, more studies using several characterization methods and public databases are needed to understand the population structure of *B. henselae*.

Although all these methods have identified a subset of *B. henselae* variants more frequently associated with human disease [7], [11], [12], [22], the number of human strains characterized is limited because *B. henselae* is extremely difficult to isolate from human samples [29]. Some of the cited methods have been applied directly to clinical samples, increasing the data available on *B. henselae* of human origin [22], [25], although, the high number of targets that have to be analyzed can hamper a full characterization, especially when the sample sizes are small. Here, we present data on the distribution of *B. henselae* variants in patients, cats and fleas from Spain. Samples were characterized by MLST and MLVA, which have been optimized to reduce the number of PCR reactions and minimize the size of sample needed. They have allowed us to fully characterize clinical samples from 46 patients, which represents the largest series of human cases characterized with MLST and MLVA to date.

## Materials and Methods

### Ethics Statement

This study and the procedures for using animal and human samples, including blood and tissue samples, were approved by the “Comité de Ética de la Investigación y de Bienestar Animal, Instituto de Salud Carlos III” (approval no. CEI PI 11-2010) in accordance with the Declaration of Helsinki and the Spanish legislation respecting the individual privacy of the patients (the entire study was conducted in Spain). Therefore, all human samples collected before 2007 were anonymized, eliminating the link between the patient’s identity and the sample, while written informed consents from the patient’s were obtained from the remaining samples in accordance with the Spanish Law of Biomedicine Research 14/2007. In addition, the animal shelter, and the owners of the cats from Madrid gave permission for their animals to be used in this study.

### Samples Analyzed in the Study

Blood samples were collected from 147 cats from an animal shelter in La Rioja (Northern Spain) in 2001 and 2002. These samples were initially collected to determine the health status of the cats. Cats were classified in three categories according to their origin: stray cats, pet cats and barnyard cats (Table 1). These two last categories corresponded to animals that lived in houses or farms, and were abandoned by their owners in the shelter. Also, 50 blood samples from pet cats who attended a veterinary clinic in Madrid (Central Spain), previously collected for diagnostic purposes, were analyzed. The fur of all the animals was examined for the presence of fleas. Any observed specimen was collected with a forceps and stored in a tube. The flea species were identified following the proper taxonomic keys [30]. Both blood samples and fleas were kept at −80°C until they were processed.

**Table 1:** Detection of *Bartonella* spp. in cats.

	La Rioja				Madrid
	Stray (n = 79)	Pet (n = 51)	Barnyard (n = 17)	Total (n = 147)	Pet (n = 50)
PCR positive	30.4% (24/79)	23.5% (12/51)	23.5% (4/17)	27.2% (40/147)	0
*B. henselae*	26.6% (21/79)	11.8% (6/51)	23.5% (4/17)	21.1% (31/147)	0
*B. clarridgeiae*	12.7% (10/79)	11.8% (6/51)	0	10.9% (16/147)	0
*Bartonella* spp.^1^	0	3.9% (2/51)	0	0.7% (2/147)	0
Co-infections	8.9% (7/79)	3.9% (2/51)	0	6.1% (9/147)	0

1 A positive hybridization signal was obtained in these specimens with the S-CHOSCA probe (common to *B. schoenbuchensis*, *B. capreoli*, *B. chomeli* and *B. birtlesii*).

A total of 58 human samples (blood, cerebrospinal fluid –CSF-, lymph node aspirates, wound exudates, biopsies, abscesses and heart valve biopsies) from 51 patients with a PCR positive result for *B. henselae* were also characterized (Table S1). These samples have been received by our reference laboratory since 1999 for diagnostic purposes, and comply with Spanish Law (Law 14/2007).

### Molecular Detection of *Bartonella* spp

DNA was extracted from the samples with the QIAamp DNA Mini kit (Qiagen, Hilden, Germany) following the manufacturer’s instructions. In the case of cat blood samples, 200 µl was processed and DNA was eluted in 100 µl. For fleas, single specimens were first pestle-crushed in 1.5 ml tubes, and extracted as above. An extraction control, consisting of 100 µl of PBS, was included every 10 samples. The extracted DNA was quantified in a NanoDrop ND-100 spectrophotometer (NanoDrop Technologies Inc. Wilmington, Delaware USA) and 200 ng was used in each PCR. In the case of flea specimens, 10 µl of the DNA extract was used. Molecular detection and species identification were performed with a PCR combined with a Reverse Line Blotting (PCR-RLB), as described previously [31], [32]. A water negative PCR control and *B. taylorii* PCR positive control (1,000 genome equivalents) were also included in each assay.

### Isolation of *Bartonella* Strains

A volume of 100 µl from each cat blood sample which was found to be *Bartonella* positive by PCR-RLB was thawed and plated in triplicate in the following media: Columbia agar plates (Oxoid, Basingstoke,UK) supplemented with 5% of defibrinated sheep blood (Oxoid), 5% of defibrinated horse blood (Oxoid) or 5% of defibrinated horse blood plus 1% v/v hemin solution [0.1% w/v hemin (Sigma-Aldrich, St. Louis, MO, USA) in 4% v/v triethanolamine (Sigma-Aldrich)]. Plates were incubated at 35°C in a moist atmosphere under 5% CO_2_ for up to 1 month. Any growth with a morphology compatible with *Bartonella* (small, gray, round colonies) was then individually sub-cultured and tested by PCR-RLB. For the latter testing, each colony was resuspended in 100 µl of sterile PBS and incubated at 100°C for 10 minutes, and 5 µl of a 1∶100 dilution was tested in each PCR reaction. Up to six *Bartonella* isolates from each blood sample were further analyzed.

### Characterization of Isolates and Positive *B. henselae* Samples

*B. henselae* strains isolated from cats, as well as samples (human, cat and flea) from which no isolates were obtained, were characterized by MLST and MLVA. In addition, nine *B. henselae* strains isolated from cats in Catalonia (strain collection of the Corporació Sanitària i Universitària Parc Taulí) were included in the study. In both methods, the Houston and Berlin-2 *s*trains were used as reference controls.

#### Characterization by MLST

MLST analysis was performed by amplifying and sequencing the eight housekeeping genes previously described [19]. As only a small amount of DNA was available from most samples, a multiplex semi-nested PCR was designed to reduce the number of reactions needed to amplify the eight targets. Therefore, in a first round, two different reaction mixtures (MLST1 and MLST2) were prepared in 50-µl final volumes. Each mixture contained the primer sets for four genes (Table S2) at a final concentration of 0.2 µM, 2×PCR mastermix of the Multiplex PCR Kit (Qiagen) and 200 ng of DNA of each sample or 10 µl in the case of flea specimens. In the case of MLST2, 5 µl of the Q solution from the Multiplex PCR Kit (Qiagen) was also added. The PCR was performed at 95°C for 15 min, followed by 40 cycles of 94°C for 30 sec, 55.5°C for MLST1 or 58.4°C for MLST2 during 90 sec, and 72°C for 90 sec, with a final extension at 72°C for 10 min. In the second round, 2 µl of each reaction was re-amplified in single PCR reactions for each housekeeping gene. All these were performed in 50-µl reaction volumes containing 10mM Tris-HCl, 50 mM KCl, 2 mM MgCl_2_, 200 µM of each deoxynucleoside triphosphate (Promega, Madison, WI, USA), 0.8 µg bovine serum albumin (Roche Diagnostics GmbH, Manheim, Germany), 1 µM of each primer (Table S2) and 1 U of *Taq* Gold polymerase (Applied Biosystems, Branchburg, NJ, USA). Cycling was the same for each target and included 94°C for 9 min, followed by 40 cycles of 94°C for 30 sec, 45°C for 30 sec (56.7°C in the case of the *groEL* and *ftsZ* targets) and 72° for 60 sec, with a final extension of 72°C for 10 min. In the case of the *B. henselae* isolates, only the second round of single PCRs was performed by adding 5 µl of the 1∶100 dilution of the boiled-extract of each strain. As multiplexing eight targets in a single tube resulted in a 10-fold reduction in sensitivity, we performed the amplification in two tubes as described above.

After amplification, PCR products were run in 1% low-melting agarose gels (Pronadisa, Torrejón de Ardoz, Spain) stained with 0.01% GelRed (Biotium Inc. Hayward, CA, USA), and the bands with the expected size were purified using the QIAquick gel extraction kit (Qiagen) and sequenced with the Big-Dye terminator cycle sequencing kit v3.1 (Applied Biosystems) following the manufacturer’s instructions. The STs were assigned according to the *B. henselae* MLST online database (http://bhenselae.mlst.net) hosted at Imperial College, UK. The alleles discovered in this study were submitted to this website to obtain new allele and ST numbers, as well as to GenBank.

#### Characterization by MLVA

MLVA was performed by studying the five VNTRs (BHV-A to BHV-E) previously described [27] with several modifications: a fluorochrome was attached at each reverse primer to perform the analysis with the GenScan technology, and two multiplex PCRs were designed to reduce the number of PCR reactions. Two reaction mixtures (VNTR1 and VNTR2) were prepared in 50-µl final volumes. These mixtures contained the primers for three and two VNTRs (Table S2), 2×PCR mastermix of the Multiplex PCR Kit (Qiagen), and 200 ng DNA of each sample or 10 µl in the case of flea specimens. In the case of the *B. henselae* isolates, 5 µl of the 1∶100 dilution of the boiled-extract of each strain was added to the mixtures. The samples were amplified at 95°C for 15 min, followed by 40 cycles of 94°C for 30 sec, 61.8°C for 90 sec, and 72°C for 90 sec, with a final extension of 72°C for 10 min. After that, 1 µl of a 1∶10 dilution of each PCR product was mixed with 0.5 µl of the labeled size standard LIZ1200 (Applied Biosystems) and 9 µl of Hi-Di™ formamide (Applied Biosystems). This mixture was incubated at 95°C for 3 min and was then separated in a POP-7 ™ polymer in a 3730×L DNA Analyzer (Applied Biosystems). The GeneScan data were subsequently analyzed with the PeakScan software v1.0 (Applied Biosystems). Fragments over 1,300 bp, which were not well resolved with our method, were separated in 0.8% MS-8 agarose gels (Pronadisa) stained with 0.01% GelRed (Biotium Inc.) for 16 hours at 50 volts. The identified profiles were submitted to a public database (http://mlva.u-psud.fr/) which has been created for this study with all the published profiles up to date. These profile numbers have been assigned chronologically according to the date of the publications where these profiles were described [12], [26], [27]. The MLVA profile 1 was assigned to the Houston strain.

### Phylogenetic Analysis

Phylogenetic inferences of the new STs identified in our study and for the 30 STs deposited in the online database were carried out by aligning the concatenated sequences of the eight targets of the MLST [without including the primer sequences (3,397–3,398 bp)] with the Multi-Alignment Fast Fourier Transform (MAFFT) method [33]. The best-fit model of nucleotide substitution was selected and a tree was built using the maximum-likelihood method [34]. The reliability of different phylogenetic groupings was evaluated by using the bootstrap test (1,000 replications).

MLVA profiles were analyzed using InfoQuest™ FP 4.50 (BioRad, Hercules, CA, USA). Clustering analysis was performed including all the 174 profiles described previously [26], [27], [35] together with the 41 from this study, using the Unweighted Pair Group Method Using Arithmetic Averages (UPGMA) to infer the phylogenetic relationships of the profiles. The minimum spanning tree with all these profiles was built with the same software.

### Statistical Data Analysis

Associations between ST and origin (region and host type) were assessed by testing their absolute frequencies with the Fisher’s exact test (FET) of the Proc Freq of the SAS statistical package (SAS Inc., Cary, NC, USA). Flea burdens in cats were submitted to analysis of variance with the Proc GLM of the SAS statistical package where the means were compared with the LSMEANS statement. Statistical significance was established at the p≤0.05 level, although exact p values are given throughout the text for each test.

## Results

### *Bartonella* spp. Detection in Cats

In La Rioja, 27.2% of the cats (40/147) were positive to *Bartonella* spp. (Table 1). *B. henselae* was identified in 21.1% of the animals (31/147) and *B. clarridgeiae* in 10.9% (16/147). In 0.7% (2/147) a reaction against the probe S-CHOSCA, common to *B. schoenbuchensis*, *B. capreoli*, *B. chomeli* and B. *birtlesii* [31], was observed (Table 1). Furthermore, the intergenic spacer 16S–23S rRNA was sequenced in one of these 2 samples, and 100% similarity with the intergenic spacer of *B. capreoli* (GenBank accession no. EU098131) was found. It is noteworthy that 6.1% of the studied cats (9/147) presented co-infections with two different *Bartonella* species (Table 1). In contrast with these results, the 50 cats from Madrid were all negative.

Prevalence of *Bartonella* spp. was significantly different (FET p<0.0001) depending on the origin of the cat (Table 1) because of lack of infection among the cats from Madrid. In La Rioja, stray cats (26.6%) and barnyard cats (23.5%) showed a non-significant higher infection rate with *B. henselae* than pet cats (11.8%, FET p = 0.1285). However, if stray and barnyard cats were taken together (26.04%) for comparison with pet cats, the difference became statistically significant (FET p = 0.0323).

### *Bartonella* spp. Detection in Fleas

A total of 62 fleas (*C. felis*) were collected from 147 cats in La Rioja (Table 2), in contrast with cats from Madrid, none of which were found to be infested. In La Rioja, the rate of infested animals ranged from 21.5% among stray cats (17/79), to 11.8% in barnyard cats (2/17) and 9.8% in pet cats (5/51), these differences were not statistically significant (FET p = 0.2090). The mean flea burden per stray cat (0.61) was also not significantly higher compared to that of pet cats (0.22, LSMEANS: p = 0.1224) and barnyard cats (0.17, LSMEANS: p = 0.9912).

**Table 2:** *Bartonella* spp. detected in fleas from La Rioja.

	Stray (n = 48)^1^	Pet (n = 11)	Barnyard (n = 3)	Total (n = 62)
PCR positive	29.2% (14/48)	0	66.7% (2/3)	25.8% (16/62)
*B. henselae*	12.5% (6/48)	0	0	9.7% (6/62)
*B. clarridgeiae*	14.6% (7/48)	0	66.7% (2/3)	14.5% (9/62)
*Bartonella* spp.^2^	2.1% (1/48)	0	0	1.6% (1/62)
Co-infections	0	0	0	0
fleas/cat^3^	0.61 (48/79)	0.22 (11/51)	0.17 (3/17)	0.42 (62/147)
fleas/parasited cat^4^	2.82 (48/17)	2.20 (11/5)	1.5 (3/2)	2.58 (62/24)

1 Number of fleas collected from all the animals of each category. The number of fleas per cat ranged from zero to six (Table S3).

2 A positive signal was obtained in this specimen with the S-CHOSCA probe (common to *B. schoenbuchensis*, *B. capreoli*, *B. chomeli* and *B. birtlesii*).

3 Mean of fleas in each cat category.

4 Mean of fleas on infested cats.

*Bartonella* spp. were detected in 25.8% of the analyzed fleas (16/62) (Table 2). The most prevalent species was *B. clarridgeiae*, detected in 14.5% of the specimens, followed by *B. henselae*, in 9.7%. This last species was only detected in fleas collected from stray cats. Also, one of the fleas was infected with a *Bartonella* which hybridized with the probe S-CHOSCA in the PCR-RLB (Table 2). Surprisingly, there was not a good correlation between positive cats and fleas infected with *Bartonella* (FET: p = 0.1304): in only three cases was the infection detected simultaneously in both stray cats and their fleas (Table S3).

### Strains Isolated from Cats from La Rioja

Sixty-nine *B. henselae* isolates were obtained from 26 of 31 *B. henselae* positive cats (83.9% of efficiency), even though the samples were cultured 10 years after they were collected. In contrast, only four *B. clarridgeiae* isolates were obtained from four of the 16 positive cats (25.0% of efficiency). These isolates were obtained in the plates supplemented with hemin. No isolates were obtained from the two positive cats to the S-CHOSCA probe.

### MLST Analysis

MLST characterization was accomplished for 78 *B. henselae* isolates (69 from La Rioja and 9 from Catalonia) obtained from 35 cats. Also, 53 human positive samples from 46 patients, four positive cat bloods from which no isolates were obtained and three positive fleas collected from two cats were fully characterized. All the strains and samples obtained from the same cat or patient presented identical ST (data not shown).

**Figure 1:**
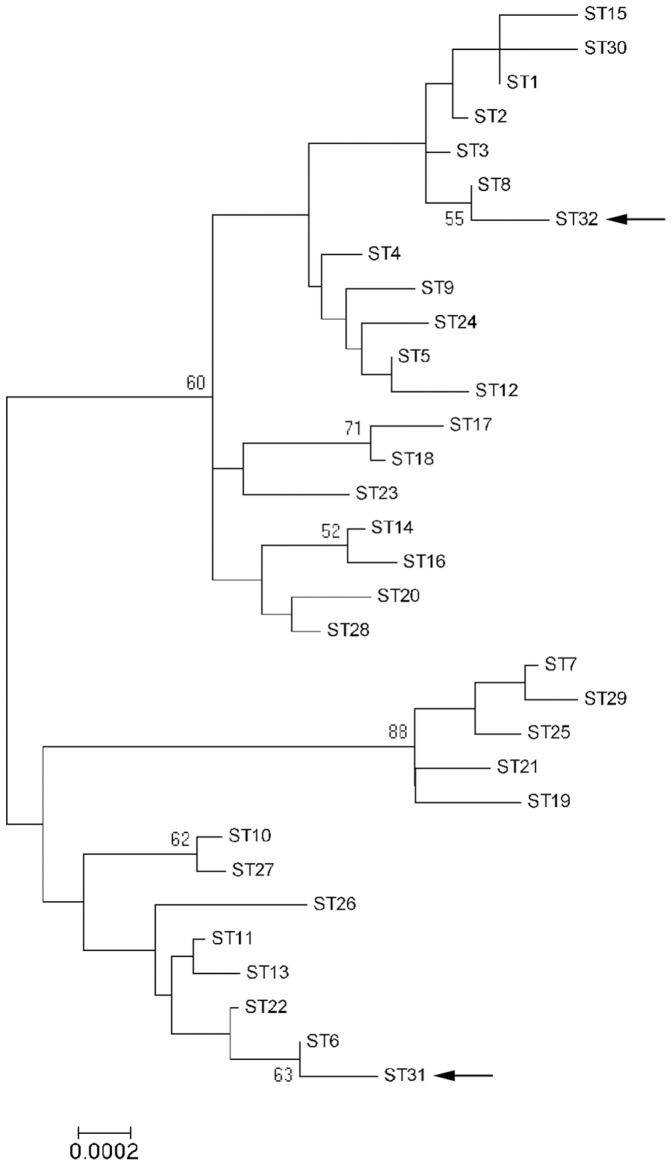
Dendrogram of the *B.* *henselae* MLST STs. Concatenate sequences of the 30 STs (3397–3398 pb) present in the MLST database (http://bhenselae.mlst.net) and the two new STs identified in this study (black arrows) were analyzed with MEGA5 software. The best nucleotide substitution model was Jukes-Cantor+I (rate for invariant sites), which was applied to build the dendrogram with the maximum likelihood method. Bootstrap values of less than 50% have been deleted from the tree. Scale bar denotes substitutions per nucleotide position.

In total, 10 different STs were found, including two new STs [ST31 (2,3,2,4,2,1,1,2) and ST32 (1,5,1,1,1,1,1,1)] after the detection of two new alleles (GenBank accession no. KC952888 for *ftsZ* allele 4, and no. KC952887 for *batR* allele 5). The phylogenetic analysis indicates that ST31 is closely related to ST6, and ST32 to ST8 (Figure 1). Both STs were found only in the feline population of La Rioja (Table 3). ST5 was the most frequent ST in both humans and felines, with a prevalence of 54.3% in patients (25/46) and 61.5% in cats (24/39) (Table 3). However, there were differences between both groups: ST8 (21.7%) and ST1 (13.0%) were overrepresented among the *B. henselae* infecting humans, while ST6 (15.4%) and ST9 (10.3%) were more frequent in cats (Table 3). This different distribution was statistically significant (FET: p = 0.00002). Interestingly, five clinical cases with a presentation of the disease different from the classical CSD, such as endocarditis, fever of unknown origin or peliosis, were associated with ST1 in a statistically significant way (FET: p = 0.0118) (Table 3). Regarding the feline population, there were no statistically significant differences in the distribution of STs among all groups (FET: p = 0.5243), although there were statistically differences in their geographical distribution (FET: p = 0.0006): ST6 (20.0% 6/30) was mainly found in La Rioja and ST9 (44.4% 4/9) mainly in Catalonia (Table 3). The three characterized positive fleas belonged to ST5 (Table 3). The individual analysis of the 16S rRNA revealed that 92.3% of the infected cats (36/39) harbored *B. henselae* GT II (Table 3). In contrast, only 54.3% of the human samples (25/46) were infected with this GT, being a statistically significant difference (FET: p = 0.0001).

**Table 3:** Distribution of the MLST STs identified in the study.

		Cats					Patients			p-value^3^	Fleas
		La Rioja			Catalonia	Total	Clinical picture		Total		
**MLST**	***rrs*** 1	**Stray**	**Pet**	**Barnyard**			**CSD**	**Others** 2			
**ST1**	**I**	0	0	0	0	0	7.3% (3/41)	60.0% (3/5)	13.0% (6/46)	**0.0289** 4	0
**ST2**	**I**	0	0	25.0% (1/4)	0	2.6% (1/39)	0	20.0% (1/5)	2.2% (1/46)	1	0
**ST3**	**I**	0	0	0	0	0	9.8% (4/41)	0	8.7% (4/46)	0.1212	0
**ST5**	**II**	71.4% (15/21)	80.0% (4/5)	50.0% (2/4)	75.0% (3/9)	61.5% (24/39)	58.4% (24/41)	20.0% (1/5)	54.3% (25/46)	0.5187	100% (3/3)
**ST6**	**II**	19.0% (4/21)	20% (1/5)	25.0% (1/4)	0	15.4% (6/39)	0	0	0	**0.0075** 4	0
**ST7**	**II**	0	0	0	11.1% (1/9)	2.6% (1/39)	0	0	0	0.4588	0
**ST8**	**I**	0	0	0	11.1% (1/9)	2.6% (1/39)	24.4%(10/41)	0	21.7% (10/46)	**0.0095** 4	0
**ST9**	**II**	0	0	0	44.4% (4/9)	10.3% (4/39)	0	0	0	**0.0406** 4	0
**ST31**	**II**	4.8% (1/21)	0	0	0	2.6% (1/39)	0	0	0	0.4588	0
**ST32**	**I**	4.8% (1/21)	0	0	0	2.6% (1/39)	0	0	0	1	0
**Total**		21	5	4	9	39	41	5	46		3

1 *rrs*: 16S rRNA genotyping.

2 Other symptoms different from CDS.

3 Fisher’s exact test performed with the frequencies of each individual STs in cats and patients.

4 Statistically significant differences.

### MLVA Analysis

The MLVA characterization was completed in the same *B. henselae* isolates and samples characterized by MLST, except for one of the patient samples (Table 4). The profiles identified in all the isolates from the same cat and all the samples from the same patient were identical, as happened with the data obtained by MLST. A total of 41 profiles were found, with only six of them previously described (Table 4). All the alleles were known, except the 11 copies of BHV-D of profile 175 (Table 4).

**Table 4:** MLVA profiles identified in the study.

MLST	MLVA						Origin		
ST	BHV-A	BHV-B	BHV-C	BHV-D	BHV-E	Profile^1^	Humans	Cats	Fleas
1	10	30	10	7	3	170	1	0	0
	10	31	10	7	3	171	1	0	0
	14	32	10	7	5	172	1	0	0
	15	20	10	8	2	86^2^	2	0	0
	15	20	10	7	4	173	1	0	0
2	15	19	10	8	2	174	1	0	0
	10	32	8	11	2	175	0	1	0
3	14	15	6	5	5	202	1	0	0
	14	25	6	5	5	203	3	0	0
5	14	26	8	7	4	167^2^	1	0	0
	14	26	10	8	4	176	0	1	0
	14	25	13	8	4	177	0	1	0
	14	25	9	1	4	178	1	0	0
	14	25	8	7	4	179^3^	1	4	0
	14	31	8	7	4	180^3^	0	4	0
	14	32	8	7	4	72^2^	11	6	2
	13	32	8	7	4	74^2^	1	0	0
	14	33	8	7	4	181	0	3	1
	14	6	8	7	4	183	1	0	0
	14	31	8	2	4	184	0	1	0
	14	32	7	7	4	185	0	1	0
	14	32	1	7	4	186	0	1	0
	14	32	3	7	4	187	1	0	0
	14	25	10	7	4	188	1	0	0
	14	32	8	2	5	189	1	1	0
	14	32	8	2	1	190	2	0	0
	14	32	8	7	3	191	0	1	0
	14	32	8	7	6	192	1	0	0
	14	32	8	7	5	193	1	0	0
	14	29	8	7	1	194	1	0	0
	13	ND^4^	ND	ND	4	ND	1	0	0
6	10	32	3	1	1	195	0	5	0
	10	26	3	1	1	196	0	1	0
7	10	14	2	2	1	27^2^	0	1	0
8	14	31	10	5	5	198	4	0	0
	14	31	6	5	5	199	4	0	0
	14	32	2	5	5	204	0	1	0
	14	31	3	5	6	201	1	0	0
	14	31	2	5	6	20^2^	1	0	0
9	14	25	8	7	4	179^3^	0	2	0
	14	23	8	7	4	182	0	1	0
	14	31	8	7	4	180^3^	0	1	0
31	10	33	3	1	1	197	0	1	0
32	14	31	6	5	6	200	0	1	0
INC^5^	INC	INC	INC	INC	INC	ND	5	1	3
					Total		51	40	6

Reference strains: Houston (ST1, MLVA profile 1∶14,20,10,7,5) and Berlin2 (ST7, MLVA profile 27∶10,14,2,2,1).

1 Profiles numbered according with the public MLVA database in http://mlva.u-psud.fr/.

2 Profiles found in other studies.

3 Identical profiles that belong to different STs.

4 ND: No determined.

5 INC: Incomplete characterization.

**Figure 2:**
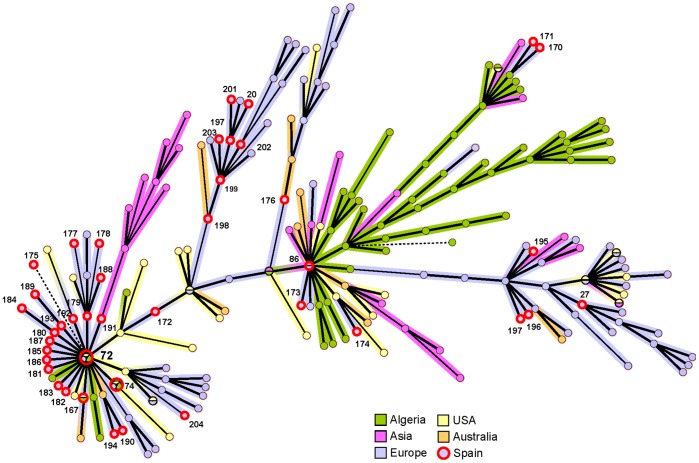
Minimum spanning tree of the *B.* *henselae* MLVA profiles. Profiles connected by a shaded background differ by a maximum of one of the five VNTR markers; regular connecting lines represent two marker differences; thick interrupted lines represent three differences. The length of each branch is also proportional to the number of differences. The colors of the branches and circles are related to the geographical origin of the detected profile. All the isolates from Spain used to build this diagram were obtained in this study. They are labeled with a thick red circle and their own profile number according to the public database (http://mlva.u-psud.fr/).

Profile 72 was highly frequent, detected in 23.9% (11/46) of the patients and 15.4% (6/39) of the cats (Table 4). Also, 50.0% (23/46) of the *Bartonella* present in human samples and 66.7% (26/39) in the feline population revealed profiles placed in clade B, where profile 72 is located (Figure S1). It is noteworthy that this profile 72 is the main founder from which most of the profiles detected in this study were generated, as is shown in the minimum spanning tree diagram (Figure 2). In addition, 30.4% of the patients (14/46) also harbored other groups of profiles which were closely related to profile 198 placed in clade C (Figure S1). Interestingly, 20.6% of the cats (8/39) were infected with profiles located in clade A (27, 195, 196 and 197), in which none of the profiles were found in patients in this or previous studies (Figure S1). Regarding fleas, the two profiles identified (72 and 181) were identical to those infecting the cats from which they were collected (Table S3).

### Geographical and Temporal Distribution

Among the profiles detected in humans, profile 72 was widely distributed, being detected in seven provinces of Spain, while others, such as profile 203, were found only in northwestern Spain (Table S1, Figure S2). In addition, some regions presented specific distributions. For example, in A Coruña, only profile 72 was detected, while in Madrid and Asturias five different profiles were identified (Table S1, Figure S2). Data for comparing profiles detected in patients and cats from the same province were limited. Human samples were not available from La Rioja where most of the feline isolates were obtained. However, profiles 72 and 189 isolated from cats in La Rioja, were also detected in two CSD cases in the neighboring province of Navarra. In Barcelona (Catalonia), only profile 72 was detected in both feline isolates and human samples (Table S1, Figure S2). It is worth pointing out that some profiles were also found in some regions over time. For example, profile 72 was detected successively in 2005, 2007 and 2011 in A Coruña, and profile 198 was identified in 2004, 2007 and 2010 in Asturias (Table S1, Figure S2).

### Discrepancies between the MLST and MLVA Methods

In this study, 10 different MLST STs were identified, whereas 41 MLVA profiles were found. Although MLVA presented a higher discriminatory capacity, in two cases (profiles 179 and 180) the same profile belonged to two different MLST STs (Table 4). In the MLVA dendrogram (Figure S1), profiles that belong to ST5 and ST9 or ST8, ST3 and ST32 were closely related. However, some profiles that belong to the same STs were placed in different clades of the dendrogram. For example: 174 and 175; 176, 177, 178 and other profiles of the ST5; or 172 and other profiles of the ST1 (Figure S1).

## Discussion

In order to know the variability of *B. henselae* in Spain, we have analyzed samples from patients, felines and fleas. We have found 27.2% of cats infected with *Bartonella* spp. in La Rioja, while in studies from other parts of Spain a lower ratio of infection was detected (4% to 6%) [36], [37]. It is noteworthy that large differences are seen worldwide in the prevalence of *Bartonella* in cats, depending on the feline population targeted [38]. In fact, stray cats always present higher percentages of infection than pet cats [38], and flea infestation correlates with bacteremia [15], [39], [40]. This is in agreement with our findings where stray cats presented higher *B. henselae* prevalence and flea infestation, although this latter parameter was not statistically significant in our study. In contrast, none of the pet cats included in this study from Madrid were infested with fleas and were all *Bartonella* negative, supporting previous studies from the same region [41], where only 0.3% of cats were found infected by *Bartonella*.

*Bartonella* spp. were detected in 25% of the fleas analyzed, which is similar to the infection rate detected in a previous study from La Rioja [42]. Although, as a whole, infected cats were associated with higher flea burdens, there was not a good individual correlation between the presence of positive fleas and bacteremic cats, as previously described [3]. A better adaptation of some *Bartonella* to its vector than to its mammalian host could help explain this finding [43].

Apart from *B. henselae* and *B. clarridgeiae*, we have detected other *Bartonella* species infecting both cats and fleas. Even though we were not able to isolate them, *B. capreoli* was identified by sequencing in one of the cat samples. This species has never been detected in cats. Nevertheless, *B. bovis,* a closely related species to *B. capreoli*, has been isolated from cats in USA [44]. Whether this is an accidental or an unusual finding with epidemiological implications remains to be confirmed in further studies.

We have found a large variability of *B. henselae* variants, identifying a total of 10 MLST STs and 41 MLVA profiles. It is noteworthy that two STs and 35 profiles have never been described. In a recent study in Algeria, co-infections of different variants in cats were frequently found [26], whereas all the isolates that we obtained from the same cat in our study were identical. The small number of isolates per cat analyzed and the probable special difficulty in culturing some variants could explain this discrepancy.

The majority of the *B. henselae* circulating in Spain were found to be closely related. Regarding the MLST characterization, ST5 was predominant in both humans (53.7%) and cats (61.5%). Interestingly, MLVA profile 72, which belongs to MLST ST5, was overrepresented, being detected in nearly 25% of the patients. This is a high frequency for a specific *B. henselae* variant, if we consider the large variability of MLVA profiles described to date and the low number of strains that has been assigned to each profile [12], [26], [27]. According to our data, profile 72 seems to have generated most of the variants found in our study, as is shown in the minimum spanning tree. Also, this profile was well distributed and persisted over time in some regions of Spain. This wide-spread profile is one of the few that has been found in different regions of the world and has been detected in both humans and cats [12], [26].

There are important geographical differences in the distribution of *B. henselae* variants. In Asia the distribution is very different from the rest of the world, regardless the characterization method used [11], [12], [23]. As shown in the minimum spanning tree, there are clusters of profiles that are found only in specific regions of the world. For example, in Spain the distribution of variants in the feline population of La Rioja and Cataluña, two regions roughly 400 km. (250 mi.) apart, was different, highlighting the need of future for additional local epidemiological studies.

The improved characterization protocols designed for this study have overcome the limitation of small sample sizes such as with fleas. For the first time, we have fully characterized the *B. henselae* variants from three positive fleas. Our protocol could also be applied to the study of other potential vectors. For example, *Ixodes ricinus* has been recently shown to be a competent vector for *B. henselae* [45], and several studies have detected *B. henselae* in questing ticks [43], [46]. Therefore, information about the *B. henselae* variants present in these arthropods could be of interest in understanding the epidemiology of bartonellosis.

Several studies have tried to identify subsets of *B. henselae* human-associated variants, although differences in the geographical distribution of the variants can affect the interpretation of the results, as has been mentioned above. In our study, there were significant differences in the distribution of variants infecting humans and cats. According to 16S rRNA, GT II was predominant in cats (92.3%), as in other countries of Europe, Oceania and North America [7], [9]–[12]. This is in contrast to the distribution in Asia, where GT I has been more frequently reported [13]–[17] or in North Africa where no significant differences between the two GTs were found [26]. Nevertheless, the frequencies of GT I and II in patients found in this study were similar to those observed in France [47], in contrast to the UK or other countries, where GT I was predominant [11], [12], [18], [22]. All these imply that 16S rRNA typing is not a reliable marker to infer epidemiology conclusions.

The different distribution of the MLST STs among humans and cats in Spain was statistically significant. In cats, ST5, ST6 and ST9 were the most frequent, while in human beings ST1, ST5 and ST8 were more frequently found. In previous studies, ST1 was the main variant associated with human infections in a worldwide strain collection [11]. However, there is a predominant distribution of ST1 in humans and felines in Asia and Australia [11], [13], [16] that could bias the importance of other STs as human pathogens in other regions of the world. In fact, in a recent study in the UK [22], ST2, ST5 and ST8 were responsible for the majority of the symptomatic human infections, similar to our results in two of the STs. On the other hand, ST4, ST6 and ST7 in the UK [22] and ST5 and ST7 in Germany [21] were the variants more frequently found in pet cats, which partially matches the predominance of ST5 and ST6 identified in cats in our study. These differences in the variants of *B. henselae* described by country could be related to either a specific geographical distribution or a higher pathogenic role of specific STs. This strengthens the need of performing regional studies to increase the knowledge on the epidemiology of *Bartonella*.

In this study, ST1 has been associated with a clinical presentation different from the CSD, although a small number of these patients were analyzed. Interestingly, in previous studies performed in France and the UK [22], [25], *B. henselae* variants detected in patients with a classical CSD were also different from the variants detected in patients with endocarditis or bacillary angiomatosis. This raises the hypothesis of an association between *B. henselae* subsets and clinical presentation, which would need to be confirmed with a larger series of cases.

Similarly to the MLST characterization, there were differences between *B. henselae* MLVA profiles infecting humans and cats. Those profiles found in our study were distributed in three main clades (A, B and C). Clade A was suggested to contain *B. henselae* variants less pathogenic for humans in a previous study [12]. After the analysis of 46 additional patients in this study, this clade remains without any human-associated profile, supporting the previous hypothesis. Whereas in the previous study only the variants carrying 14 and 15 copies of BVH-A were associated with humans [12], here some profiles detected in the Spanish patients contained a different number (10 and 13 copies). This indicates that BHV-A is not a good marker for identifying isolates which can infect humans. Overall, MLVA profiles belonging to the same MLST ST were closely related, although a few exceptions regarding profiles that belonged to ST1, ST2 and ST5 were found. Nevertheless, different subtypes have been recently described for some MLST STs, suggesting a larger complexity in the MLST classification [48] that could explain those exceptions.

The characterization of the different variants of *B. henselae* is important to understanding the biology of this pathogen and to evaluate its risk for public health. Among the characterization methods, MLVA has shown a higher discriminatory power than MLST, although some discrepancies have been found. In addition, MLVA has been optimized with our protocol and is easier to perform than MLST. Therefore MLVA could replace MLST for epidemiological purposes, and MLST could be used for phylogeny, as has been suggested [26].

### Conclusions

We have studied the diversity of *B. henselae* circulating in humans, reservoirs and vectors in Spain. We have improved the amplification conditions to perform MLST and MLVA directly from clinical samples as no isolates are available in the majority of cases. The improved amplification conditions have allowed us to characterize the *B. henselae* variants present in 46 patients, 39 cats and three fleas. This represents the largest series of human cases characterized by these methods to date and the first report from vectors.

We have found a large variability of *B. henselae* variants in Spain. Overall we have identified 10 MLST STs and 41 MLVA profiles. Among these, two STs and 35 profiles were not previously described. Also, the majority of the variants from this study were closely related. In fact, ST5 was the predominant variant infecting felines and human beings. Among all the *B. henselae* variants which belong to the ST5, we have also identified a common MLVA profile (72). This profile is well distributed in Spain, persists over time, and seems also to have generated most of the variants identified in this study. In addition, we have identified some variants that were more frequently found infecting humans and others found only in cats. Interestingly, some of these latter variants were located in a separate clade, and could represent strains less pathogenic for humans, as has been previously hypothesized.

## Supporting Information

Figure S1 **MLVA profile dendrogram.** A dendrogram was built with the profiles identified in this study and those described previously. The clades in which the profiles identified in this study were found are framed and labeled A, B and C.
(PDF)

Figure S2 **Geographical distribution of profiles detected in human samples.** Each profile identified in the 46 human samples is assigned to the Spanish province of origin. The color of each circle represents the MLST ST of the sample and the number inside the circle corresponds to the MLVA profile number.
(TIF)

Table S1 **Patients analyzed in this study.**
(DOCX)

Table S2 **Primers used for MLST and MLVA characterization.**
(DOCX)

Table S3 **Summary of data of cats infested by fleas.**
(DOCX)
