# Supplementary figures and images for: Distribution of Bartonella henselae Variants in Patients, Reservoir Hosts and Vectors in Spain
Source: PLoS One. 2013 Jul 9;8(7):e68248. doi: 10.1371/journal.pone.0068248 (PMC3706593; doi:10.1371/journal.pone.0068248)

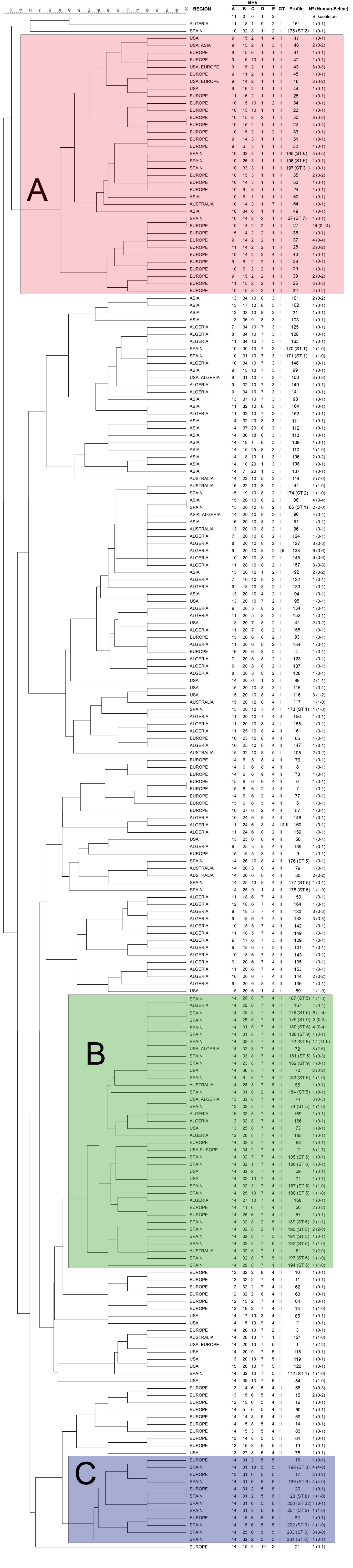

Supplement: Figure S1 — MLVA profile dendrogram. A dendrogram was built with the profiles identified in this study and those described previously. The clades in which the profiles identified in this study were found are framed and labeled A, B and C. (PDF) [file pone.0068248.s001.pdf]

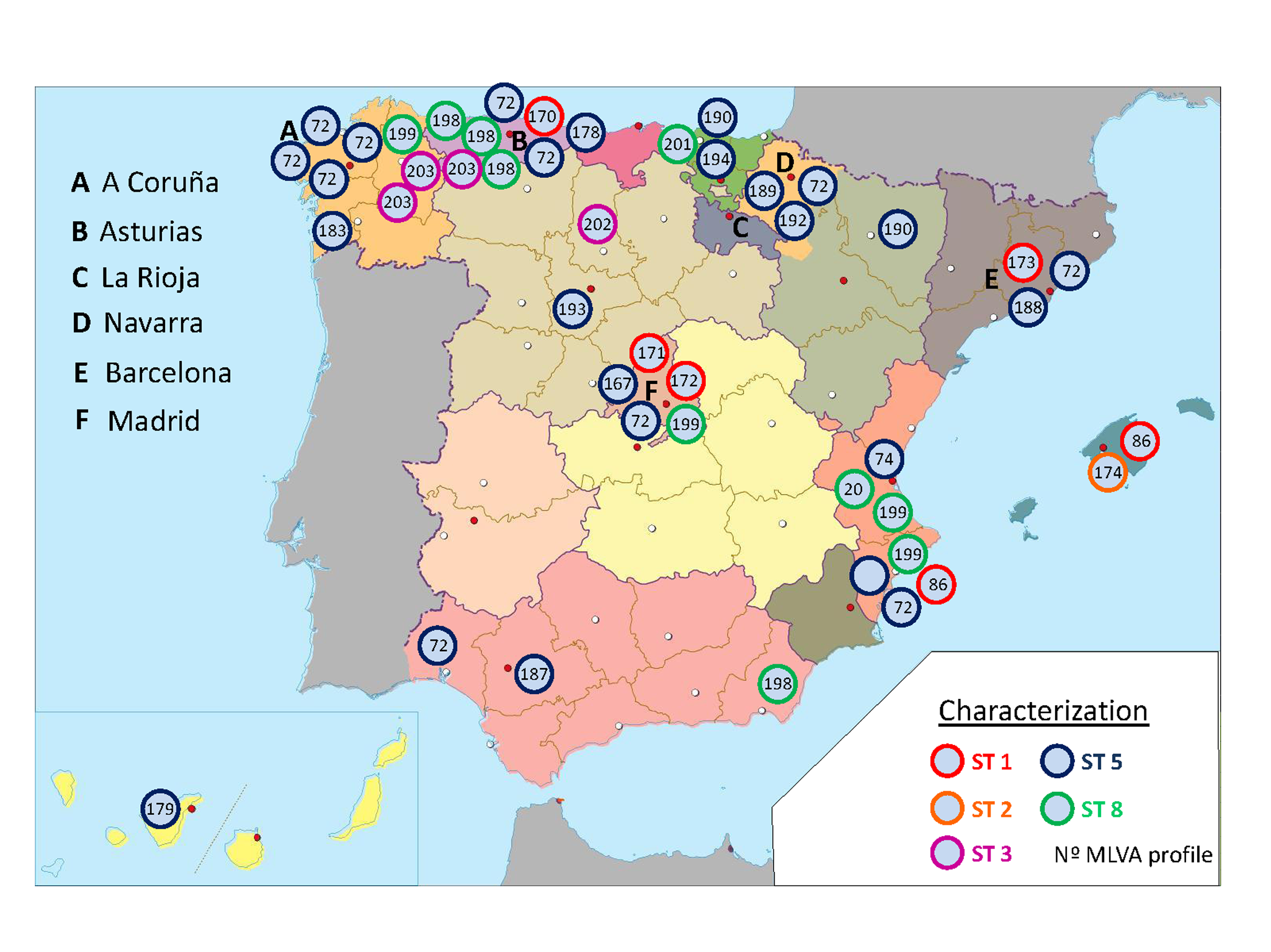

Supplement: Figure S2 — Geographical distribution of profiles detected in human samples. Each profile identified in the 46 human samples is assigned to the Spanish province of origin. The color of each circle represents the MLST ST of the sample and the number inside the circle corresponds to the MLVA profile number. (TIF) [file pone.0068248.s002.tif]
