# Supplementary material for: Distribution of Bartonella henselae Variants in Patients, Reservoir Hosts and Vectors in Spain
Source: PLoS One. 2013 Jul 9;8(7):e68248. doi: 10.1371/journal.pone.0068248 (PMC3706593; doi:10.1371/journal.pone.0068248)
Supplement: Table S1 — Patients analyzed in this study. (DOCX) [file pone.0068248.s003.docx]

**Table S1. Patients analyzed in this study.**

| **Patient** | **Gender** | **Age** | **Sample** | **Clinical picture^1^** | **Province** | **Year** | **MLVA^2^** | **MLST^3^** |
| --- | --- | --- | --- | --- | --- | --- | --- | --- |
| 1 | Male | 8 | Lymph node exudate | CSD | Alicante | 2005 | INC^4^ | 5 |
| 2 | Female | 40 | Lymph node exudate | CSD | Alicante | 2006 | 199 | 8 |
| 3 | Female | 44 | Lymph node aspirated | CSD | Alicante | 2008 | 72 | 5 |
| 4 | Male | 75 | Blood | FUO | Alicante | 2011 | 86 | 1 |
| 5 | Male | 25 | Lymph node exudate | CSD | Almería | 2005 | 198 | 8 |
| 6 | Female | 18 | Lymph node biopsy | CSD | Asturias | 2004 | 198 | 8 |
| 7 | Male | 35 | Lymph node biopsy | CSD | Asturias | 2006 | 170 | 1 |
| 8 | Male | 8 | Lymph node biopsy | CSD | Asturias | 2007 | 198 | 8 |
| 9 | Male | unknown | Lymph node exudate | CSD | Asturias | 2007 | 72 | 5 |
| 10 | Male | 14 | Lymph node aspirated | CSD | Asturias | 2009 | 203 | 3 |
| 11 | Male | 30 | Lymph node exudate | CSD | Asturias | 2010 | 198 | 8 |
| 12 | Male | 20 | Cutaneus abcess | CSD | Asturias | 2012 | 72 | 5 |
| 13 | Male | 78 | Valve biopsy | CNEC | Asturias | 2012 | 178 | 5 |
| 14 | Male | unknown | Valve biopsy | CNEC | Baleares | 2006 | 174 | 2 |
| 15 | Male | unknown | Valve biopsy | CNEC | Baleares | 2006 | 86 | 1 |
| 16 | Male | 39 | Lymph node biopsy | CSD | Cádiz | 2010 | 72 | 5 |
| 17 | Male | 52 | Lymph node biopsy | CSD | Barcelona | 2010 | 173 | 1 |
| 18 | Male | 10 | Lymph node biopsy | CSD | Barcelona | 2011 | 72 | 5 |
| 19 | Female | 50 | Lymph node aspirated | CSD | Barcelona | 2009 | 188 | 5 |
| 20 | Male | 48 | Lymph node aspirated | CSD | Huesca | 2012 | 190 | 5 |
| 21 | Male | 28 | Lymph node biopsy | CSD | A Coruña | 2005 | 72 | 5 |
| 22 | Female | 7 | Lymph node exudate | CSD | A Coruña | 2005 | 72 | 5 |
| 23 | Male | 53 | Lymph node exudate | CSD | A Coruña | 2007 | 72 | 5 |
| 24 | Female | 45 | Lymph node biopsy | CSD | A Coruña | 2011 | 72 | 5 |
| 25 | Female | 55 | Lymph node exudate | CSD | Lugo | 2005 | 203 | 3 |
| 26 | Male | 56 | Lymph node exudate | CSD | Lugo | 2008 | 203 | 3 |
| 27 | Female | 56 | Lymph node biopsy | CSD | Lugo | 2011 | 199 | 8 |
| 28 | Female | unknown | Lymph node aspirated | CSD | Madrid | 1999 | 199 | 8 |
| 29 | Male | 22 | Lymph node exudate | CSD | Madrid | 2007 | 72 | 5 |
| 30 | Male | unknown | Hepatic abcess | HP | Madrid | 2008 | 172 | 1 |
| 31 | Male | 40 | Lymph node exudate | CSD | Madrid | 2008 | 167 | 5 |
| 32 | Female | 6 | Lymph node exudate | CSD | Madrid | 2011 | 171 | 1 |
| 33 | Male | 31 | Lymph node aspirated | CSD | Navarra | 2006 | 72 | 5 |
| 34 | Female | 43 | Lymph node exudate | CSD | Navarra | 2007 | 189 | 5 |
| 35 | Male | 68 | Lymph node biopsy | CSD | Navarra | 2008 | 192 | 5 |
| 36 | Female | 11 | Lymph node aspirated | CSD | Palencia | 2006 | 202 | 3 |
| 37 | Female | 35 | Lymph node exudate | CSD | Pontevedra | 2010 | 183 | 5 |
| 38 | Male | 46 | Lymph node exudate | CSD | Sevilla | 2012 | 187 | 5 |
| 39 | Male | 6 | Lymph node biopsy | CSD | Tenerife | 2005 | 179 | 5 |
| 40 | Male | 11 | Lymph node exudate | CSD | Valencia | 2002 | 20 | 8 |
| 41 | Female | unknown | Cutaneus abcess | CSD | Valencia | 2008 | 199 | 8 |
| 42 | Male | 34 | Lymph node aspirated | CSD | Valencia | 2008 | 74 | 5 |
| 43 | Male | 14 | Lymph node biopsy | CSD | Valladolid | 2012 | 193 | 5 |
| 44 | Male | 28 | Lymph node biopsy | CSD | Vizcaya | 2006 | 190 | 5 |
| 45 | Male | 46 | Lymph node exudate | CSD | Vizcaya | 2008 | 201 | 8 |
| 46 | Male | 36 | Lymph node exudate | CSD | Vizcaya | 2009 | 194 | 5 |
| 47 | Male | 8 | Lymph node biopsy | CSD | Alicante | 2001 | INC | INC |
| 48 | Female | 2 | Skin biopsy | CSD | Navarra | 2007 | INC | INC |
| 49 | Female | 39 | LCR | MG | Madrid | 2009 | INC | INC |
| 50 | Male | 49 | Blood | BA | Málaga | 2010 | INC | INC |
| 51 | Female | 35 | Lymph node biopsy | CSD | Alicante | 2012 | INC | INC |

^1^CSD: Cat Scratch Disease. Patients that presented the classical form of the disease, with enlarged lymph nodes; FUO: Fever of Unknown Origin; CNEC: Culture Negative endocarditis; HP: Hepatic Peliosis; BA: Bacillar Angiomatosis; MG: Meningitis;

^2^MLVA: profiles identified in the patients.

^3^MLST: ST identified in the patients.

^4^INC: Incomplete characterization.
