# Supplementary material for: Distribution of Bartonella henselae Variants in Patients, Reservoir Hosts and Vectors in Spain
Source: PLoS One. 2013 Jul 9;8(7):e68248. doi: 10.1371/journal.pone.0068248 (PMC3706593; doi:10.1371/journal.pone.0068248)
Supplement: Table S2 — Primers used for MLST and MLVA characterization. (DOCX) [file pone.0068248.s004.docx]

**Table S2. Primers used for MLST and MLVA characterization.**

| **Method** | **Target** | **Primer** | **Sequence 5’ to 3’** | **Master Mix^1^** | **Conc^2^** | **Reference** |
| --- | --- | --- | --- | --- | --- | --- |
| MLST | *rrs* | JI16SF | AGAGTTTGATCCTGGYTCAG | SP, MLST2 | 1; 0.2 | [7] |
|  |  | JI16SR | CTTTACGCCCARTAAWTCCG | SP | 1 | [7] |
|  |  | NES16SR | CCCTGGGATTTCACCTCTG | MLST2 | 0.2 | This study |
|  | *batR* | JIbatRF | GACCGCAATATTTTGACATC | SP | 1 | [19] |
|  |  | JIbatRR | GCATCCATCAAAGCATCACGACTT | SP, MLST 2 | 1; 0.2 | [19] |
|  |  | NESbatRF | CGCAATATTTTGACATCGTTATCT | MLST 2 | 0.2 | This study |
|  | *gltA* | JIgltAF | GGGGACCAGCTCATGGTGG | SP | 1 | [48] |
|  |  | JIgltAR | AATGCAAAAAGAACAGTAAACA | SP, MLST2 | 1; 0.2 | [48] |
|  |  | NESgltAF | CAGGTTCATCAGGTGCTAATC | MLST2 | 0.2 | This study |
|  | *ftsZ* | JIftsZF | GCCTTCTCATCCTCAACTTC | SP, MLST 1 | 1; 0.2 | [19] |
|  |  | JIftsZR | CTTTGTTTTAAACGCTGCC | SP | 1 | [19] |
|  |  | HftsZR | TTGAACAAGTGCCCGAGAAT | MLST 1 | 0.2 | This study |
|  | *groEL* | JIgroF | GTTGATGATGCCTTGAAC | SP | 1 | [19] |
|  |  | JIgroR | TGGTGTGTCTTTCTTTGG | SP, MLST 1 | 1; 0.2 | [19] |
|  |  | NESgroF | TTGCTAAACTCGCTGGAG | MLST 1 | 0.2 | This study |
|  | *nlpD* | JInlpDF | GGCGCTGGTATGATACAA | SP | 1 | [19] |
|  |  | JInlpDR | GACATCTGTGCGGAAGAA | SP, MLST 2 | 1; 0.2 | [19] |
|  |  | NESnlpDF | TCTGGTATGCAGCGTTTC | MLST 2 | 0.2 | This study |
|  | *ribC* | JIribCF | AGCGAGGATCAAAACAAC | SP | 1 | [19] |
|  |  | JIribCR | GCTCTTCAACACAATTAACG | SP, MLST 1 | 1; 0.2 | [19] |
|  |  | NESribCF | CTTAGAAATTGGTGCATCA | MLST 1 | 0.2 | This study |
|  | *rpoB* | JIrpoBF | CGTGACGTACATCCTACA | SP | 1 | [19] |
|  |  | JIrpoBR | AACAGCAGCTCCTGAATC | SP, MLST 1 | 1; 0.2 | [19] |
|  |  | HrpoBF | TGCCACAGGATTTGATTAAC | MLST 1 | 0.2 | This study |
| MLVA | VNTR A | BHV-AF | AAATCAACACTCTCAAAAACACAAG | VNTR 1 | 0.2 | [27] |
|  |  | BHV-AR | FAM-TGCTCTGCTTCGTTTGCTCA^3^ | VNTR 1 | 0.2 | This study |
|  | VNTR B | BHV-BF | AGGAACGCTCTTACGAACTT | VNTR 1 | 0.6 | [27] |
|  |  | BHV-BR | NED-AAAATACTTCGGAGGAGGGT | VNTR 1 | 0.6 | This study |
|  | VNTR C | BHV-CF | ATTCCTGAGACCTTAGTGATT | VNTR 1 | 0.4 | [27] |
|  |  | BHV-CR | VIC-GGTGATAAAGCATTCCATAA | VNTR 1 | 0.4 | This study |
|  | VNTR D | BHV-DF | GGGAAAGCGAGTATTGAG | VNTR 2 | 0.4 | [27] |
|  |  | BHV-DR | FAM-CCCACTCTTTTTGTTATCTTC | VNTR 2 | 0.4 | This study |
|  | VNTR E | BHV-EF | CCCCACAAAAGCAATAATC ^3^ | VNTR 2 | 0.2 | This study |
|  |  | BHV-ER | NED-TGCTGATAGTGCGGTTTTC | VNTR 2 | 0.2 | This study |

^1^ Primers were mixed in reactions for single PCR (SP) or multiplex PCR of different housekeeping genes (MLST 1 and 2) or VNTRs (VNTR 1 and 2).

^2^ Final primer concentrations (µM) in the reactions (single PCR; multiplex PCR).

^3^ These primers have an extra nucleotide compared to the sequence published by Monteil et al. 2007 [27], which presented mismatches in the BLAST analysis with sequences deposited in the GenBank.
