# Supplementary material for: Distribution of Bartonella henselae Variants in Patients, Reservoir Hosts and Vectors in Spain
Source: PLoS One. 2013 Jul 9;8(7):e68248. doi: 10.1371/journal.pone.0068248 (PMC3706593; doi:10.1371/journal.pone.0068248)
Supplement: Table S3 — Summary of data of cats infested by fleas. (DOCX) [file pone.0068248.s005.docx]

**Table S3. Summary of data of cats infested by fleas.**

| Cats | | | Fleas | | | Profile^1^ |
| --- | --- | --- | --- | --- | --- | --- |
| Code | Category | PCR/RLB | Number | Positive | PCR/RLB |  |
| 1 | Stray | No | 6 | 1 | *Bartonella* spp. |  |
| 2 |  | No | 6 | 0 |  |  |
| 3 |  | No | 5 | 3 | *B. clarridgeiae* |  |
| 4 |  | No | 3 | 0 |  |  |
| 5 |  | No | 3 | 1 | *B. clarridgeiae* |  |
| 6 |  | No | 3 | 0 |  |  |
| 7 |  | No | 3 | 0 |  |  |
| 8 |  | *B. henselae* | 3 | 3 | *B. henselae* | 72 (fleas and cat)^2^ |
| 9 |  | No | 3 | 0 |  |  |
| 10 |  | No | 3 | 2 | *B. clarridgeiae* |  |
| 11 |  | No | 2 | 0 |  |  |
| 12 |  | No | 2 | 0 |  |  |
| 13 |  | No | 2 | 2 | *B. henselae* | INC (fleas)^3^ |
| 14 |  | *B. clarridgeiae* | 1 | 1 | *B. clarridgeiae* |  |
| 15 |  | *B. henselae* | 1 | 1 | *B. henselae* | 181 (both) |
| 16 |  | No | 1 | 0 |  |  |
| 17 |  | No | 1 | 0 |  |  |
| 18 | Pet | No | 3 | 0 |  |  |
| 19 |  | No | 3 | 0 |  |  |
| 20 |  | No | 2 | 0 |  |  |
| 21 |  | No | 2 | 0 |  |  |
| 22 |  | No | 1 | 0 |  |  |
| 23 | Barnyard | No | 2 | 2 | *B. clarridgeiae* |  |
| 24 |  | *B. henselae* | 1 | 0 |  | 72 (cat) |
| Total |  |  | 62 | 16 |  |  |

^1^ The MLVA profile identified in the sample is showed. The specimen from which the characterization has performed is indicated in brackets.

^2^ One of the three fleas was incomplete characterized.

^3^ INC: incomplete characterization.
